# Fgl2 regulates Fc**γ**RIIB^+^CD8^+^ T cell responses during infection

**DOI:** 10.1172/jci.insight.186259

**Published:** 2025-04-08

**Authors:** Anna B. Morris, Max W. Adelman, Kelsey B. Bennion, Catherine D. Martinez, Kem-Maria McCook, Michael H. Woodworth, Charles R. Langelier, Nadine Rouphael, Christopher D. Scharer, Cheryl L. Maier, Colleen S. Kraft, Mandy L. Ford

**Affiliations:** 1 Department of Surgery and Emory Transplant Center and; 2 Department of Medicine, Division of Infectious Diseases, Emory University, Atlanta, Georgia, USA.; 3 Department of Medicine, Division of Infectious Diseases, University of California, San Francisco, California, USA.; 4 Department of Microbiology and Immunology and; 5 Department of Pathology and Laboratory Medicine, Emory University, Atlanta, Georgia, USA.

**Keywords:** Immunology, Adaptive immunity, T cells

## Abstract

While the inhibitory receptor FcγRIIB has been shown to be upregulated on activated CD8^+^ T cells in both mice and humans, its effect on T cell fate during infection has not been fully elucidated. We identified an increase in FcγRIIB-expressing CD8^+^ T cells in patients with COVID-19 relative to healthy controls as well as in mouse models of viral infection. Despite its well-known role as an Fc receptor, FcγRIIB also ligates the immunosuppressive cytokine Fgl2, resulting in CD8^+^ T cell apoptosis. Both chronic LCMV infection in mice and COVID-19 in humans resulted in a significant increase in plasma Fgl2. Transfer of CD8^+^ T cells into a Fgl2-replete, but not Fgl2-devoid, environment resulted in elimination of FcγRIIB^+^, but not FcγRIIB^–^, CD8^+^ T cells. Similarly, plasma Fgl2 was directly proportional to CD8^+^ T cell lymphopenia in patients with COVID-19. RNA-Seq analysis demonstrated that *Fgl2* was produced by murine virus–specific CD8^+^ T cells, with an increase in *Fgl2* in CD8^+^ T cells elicited during chronic versus acute viral infection. *Fgl2* was also upregulated in CD8^+^ T cells from patients with COVID-19 versus healthy controls. In summary, CD8^+^ T cell production of Fgl2 during viral infection underpinned an FcγRIIB-mediated loss of CD8^+^ T cell immunity in both mice and humans.

## Abstract 2

Fgl2, an immunosuppressive cytokine that is significantly increased in the blood of COVID patients, induces T cell attrition via binding of the inhibitory receptor FcγRIIB.

## Introduction

T cell responses are composed of a tightly regulated series of cellular events — including activation, proliferation, effector function, and death — that can lead to either effective clearance of the infecting agent or, in some cases, an unproductive, dysregulated response characterized by persistent inflammation, exhaustion, deletion, and ineffective pathogen control (1). Identifying the distinct cellular and molecular interactions that underlie these divergent outcomes of T cell activation remains an important question in the field.

One such pathway that contributes to the regulation of CD8^+^ T cell responses during immune responses to cancer and transplantation is the inhibitory receptor FcγRIIB (2–5). We and others have recently showed that this Fc-binding, ITIM-containing receptor is upregulated on a subset of highly differentiated, multipotent CD8^+^ T cells in both mice and humans (5–9). This finding was noteworthy because Fc receptors were not previously known to be expressed on T cells. Moreover, pharmacologic blockade or genetic deletion of FcγRIIB specifically on CD8^+^ T cells resulted in an increased and more effective CD8^+^ T cell response, demonstrating that FcγRIIB functions physiologically as an inhibitory pathway to restrain CD8^+^ T cell responses (5–7). Transcriptomic analysis of FcγRIIB^+^ versus FcγRIIB^–^ CD8^+^ T cells revealed that the presence of FcγRIIB conferred a gene expression signature consistent with increased apoptosis (5), a finding that was confirmed by the identification of increased caspase 3/7 activity in FcγRIIB^+^ versus FcγRIIB^–^ CD8^+^ T cells. Intriguingly, however, these studies showed that IgG is not the only functional ligand for FcγRIIB on CD8^+^ T cells (5). Instead, our work identified a critical role for the immunosuppressive cytokine fibrinogen-like 2 (Fgl2) in binding to FcγRIIB expressed on CD8^+^ T cells and inducing the caspase 3/7–dependent apoptosis of those cells (5). In this way, Fgl2 ligation of FcγRIIB was shown to diminish the CD8^+^ T cell response.

Fgl2 was identified in 2001 and is a 439–amino acid type II integral membrane protein that contains a carboxy-terminal fibrinogen-related domain and functions in vitro and in vivo as a serine protease directly cleaveing prothrombin to produce thrombin (10). Subsequent studies revealed that in its secreted form, Fgl2 can also function as an antiinflammatory cytokine that inhibits proliferation of CD3^+^ T cells in vitro. Moreover, T cells isolated from *Fgl2^–/–^* mice have been shown to be hyper-proliferative (11). Additionally, Fgl2 can inhibit the maturation of immature DCs by preventing NF-κB translocation to the nucleus and subsequent expression of CD80 and MHC II (12). The secreted form of Fgl2 is produced by many immune cell types including Foxp3^+^ Treg (13) and has been shown to ligate FcγRIIB on DCs and macrophages (12, 14). However, a direct role for Fgl2 in controlling CD8^+^ T cell responses via FcγRIIB during viral infection has not been investigated. Here, we assess the expression of FcγRIIB in patients infected with SARS-CoV-2 and in a mouse model of LCMV. Results indicate that increased serum concentrations of Fgl2 underlie diminished FcγRIIB^+^CD8^+^ T cell responses in both mouse and human, highlighting the potential importance of this pathway in regulating CD8^+^ T cell immunity to infection.

## Results

### Increase in FcγRIIB-expressing CD8^+^ T cells in patients with COVID-19 relative to healthy controls.

**Figure 1:**
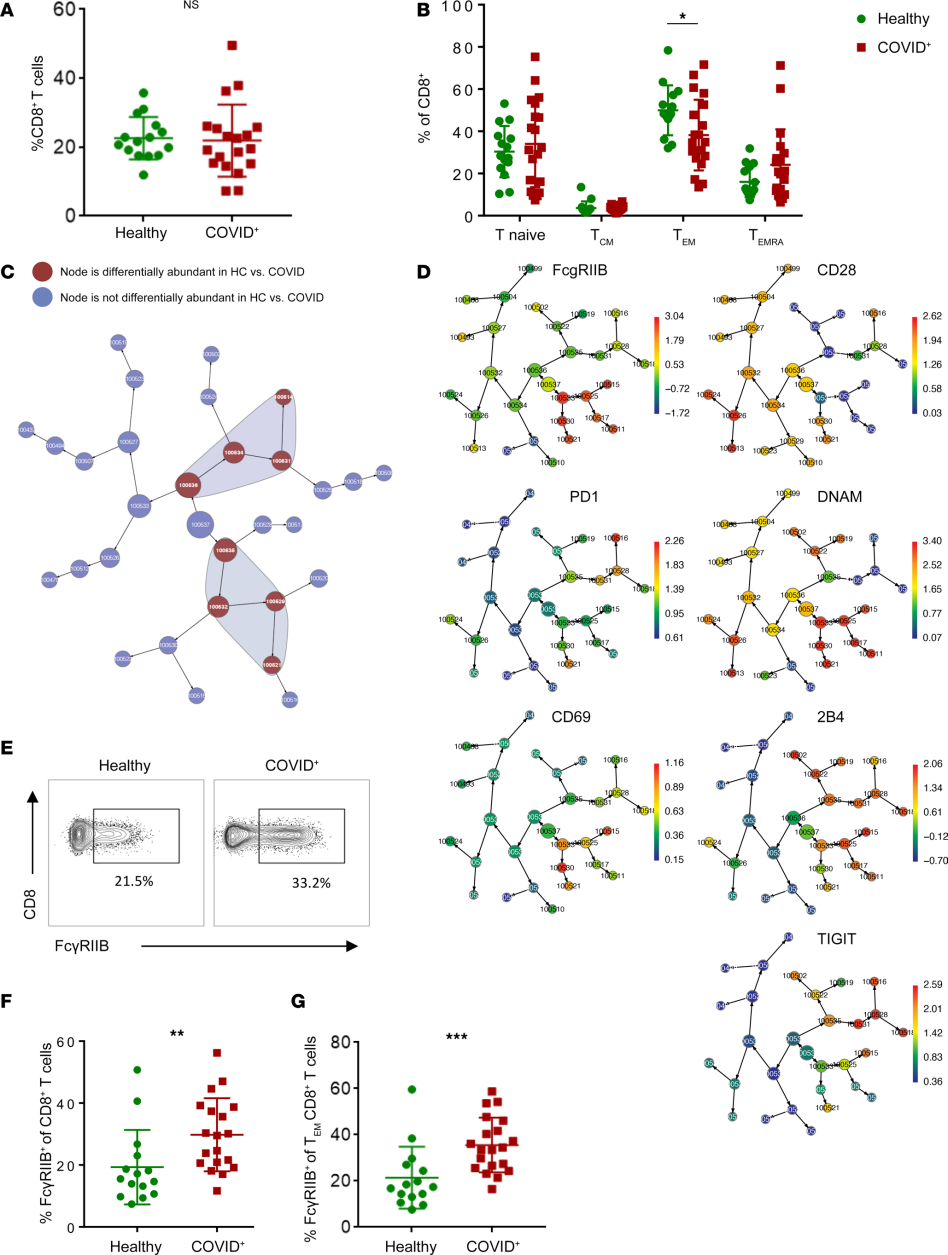
FcγRIIB is upregulated on CD8^+^ T cells isolated from patients with COVID. Patients testing positive for SARS CoV-2 admitted as inpatients at Emory University Hospital from May to July 2020 (*n* = 31; Table 1) and normal healthy controls (*n* = 15) were consented for blood draw and CD8^+^ T cells were analyzed by flow cytometry. (**A**) Bulk CD8^+^ T cell frequencies were analyzed by Mann-Whitney *U* nonparametric test. (**B**) Frequencies of naive (CCR7^+^CD45RA^+^), TCM (CCR7^+^CD45RA^–^), TEM (CCR7^–^CD45RA^–^), and TEMRA (CCR7^–^CD45RA^+^) cells among CD8^+^ T cells are shown (analyzed by 2-way ANOVA). (**C** and **D**) Flow cytometry data were subjected to spanning-tree progression analysis for density-normalized events (SPADE) analysis. Shaded areas represent clusters of cells that were differentially represented in healthy individuals versus patients with COVID. Phenotypic characteristics of cells in those clusters are shown in **D**. Intensity of expression (MFI) is indicated by colorimetric scale. (**E**–**G**) Flow cytometry data were analyzed via FlowJo. (**E**) Representative flow cytometry staining of FcγRIIB^+^ cells among CD8^+^ T cells. (**F**) Summary data of FcγRIIB^+^ cells among CD8^+^ cells (Mann-Whitney *U* nonparametric test). (**G**) Summary data of FcγRIIB^+^ cells among CD8^+^ Tem (Mann-Whitney *U* nonparametric test). Data are depicted as mean ± SEM. *P < 0.05, **P < 0.01, ***P < 0.001.

Because our previous studies in transplantation and tumor models demonstrated that signaling through FcγRIIB results in apoptosis in CD8^+^ T cells in a cell-autonomous manner, here we sought to determine the expression of FcγRIIB on CD8^+^ T cells isolated from patients hospitalized with SARS-CoV-2 infection. Patients testing positive for SARS-CoV-2 admitted as inpatients at Emory University Hospital May–July 2020 (*n* = 31; Table 1) and normal healthy controls (*n* = 15) were enrolled in an IRB-approved protocol and consented for blood draw. There were no significant differences in age, sex, race, or ethnicity between the healthy controls and patients positive for SARS-CoV-2. Blood samples were obtained 2-16 (mean 8.9) days after symptom onset and CD8^+^ T cells were analyzed by flow cytometry (Figure 1A). Results indicated a reduced frequency of CD45RA^–^ CCR7^–^ effector memory T (Tem) cells in patients with COVID-19 relative to healthy controls (Figure 1B), a finding that is consistent with other published reports (15–17). To identify additional characteristics of CD8^+^ T cell populations that were significantly different between healthy versus COVID-19, unsupervised analysis using spanning-tree progression analysis for density-normalized events (SPADE) was conducted. Bulk CD8^+^ T cells within both healthy controls (HC) and patients with COVID-19 were clustered into distinct nodes based on similarity in phenotypic markers, and the abundance of cells within each node was compared in HC versus COVID-19. Nodes containing cells that did not differ in abundance between HC versus COVID-19 are shown in blue (Figure 1C). Two clusters of cells were identified that were differentially abundant in HC versus patients with COVID-19 (Figure 1C, red nodes in shaded polygons). The intensity of staining of each phenotypic marker on cells within these differentially abundant nodes is mapped in Figure 1D and revealed a subset of CD8^+^ T cells expressing the inhibitory receptor FcγRIIB (top left panel).

These findings were verified via traditional flow cytometry analysis using FlowJo, which illuminated a significant increase in FcγRIIB^+^ cells among total CD8^+^ T cells (Figure 1, E and F) as well as a significant increase in FcγRIIB^+^ cells among CD8^+^CD45RA^–^CCR7^–^ Tem in samples isolated from patients with COVID-19 as compared with healthy controls (*P* < 0.001; Figure 1G). This increase was observed across age groups (26–40, 41–60, and 61–89 years old) (Supplemental Figure 1A; supplemental material available online with this article; https://doi.org/10.1172/jci.insight.186259DS1) and in both patients with COVID-19 who stayed in the ICU and those who did not (Supplemental Figure 1B). There was no difference in the frequency of FcγRIIB^+^ cells among CD8^+^CD45RA^–^CCR7^–^ Tem between male versus female patients with COVID-19 (Supplemental Figure 1C). Of note, the anti-FcγRIIB clone FLI8.26 used in these experiments, like many anti-FcR antibodies, has the potential cross-react and detect FcγRIIA and/or FcγIIC on myeloid cells (18). However, using quantitative PCR (qPCR) analysis of sorted FLI8.26^+^ CD8^+^ T cells, we have previously shown that the FLI8.26 staining on CD8^+^ T cells is indicative of FcγRIIB expression (19). Taken together, these results demonstrate that the inhibitory receptor FcγRIIB is upregulated on CD8^+^ T cells in patients experiencing SARS-CoV-2 viral infection.

### Virus-specific CD8^+^ T cells acquire expression of FcγRIIB in mouse models of acute and chronic viral infection.

**Figure 2:**
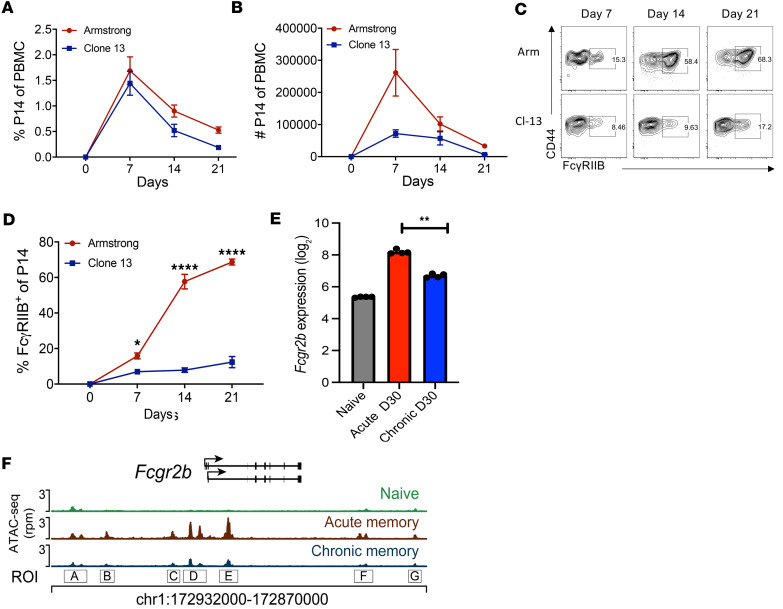
FcγRIIB is upregulated on viral antigen-specific effector and memory CD8^+^ T cells in mice. Naive B6 mice were adoptively transferred with 2 × 10^3^ Thy1.1^+^ P14 TCR tg T cells and were infected with either LCMV Arm (2 × 10^5^ pfu i.p.) or cl-13, 2 × 10^6^ pfu i.v.). Blood was collected at the indicated time points for flow cytometric analysis. (**A**) Frequencies of Thy1.1^+^ P14 among total PBMC in Arm- or cl-13–infected animals. (**B**) Absolute numbers of Thy1.1^+^ P14 within PBMC in Arm- or cl-13–infected animals. (**C** and **D**) Representative flow cytometry staining (**C**) and summary data (**D**) of FcγRIIB^+^ cells among Thy1.1^+^ CD8^+^ T cells. Data shown are *n* = 3–5/group and are representative of 2 independent experiments. Data at each time point were compared by Mann-Whitney *U* nonparametric test. (**E**) mRNA transcript levels of *Fcgr2b* RNA isolated from P14 cells from Arm-infected versus cl-13–infected mice on day 30 from an existing dataset (20) (GSE30341) were compared by 2-way ANOVA with Dunnett’s post hoc test. Naive P14 were included as a control. (**F**) Chromatin accessibility of the *Fcgr2b* locus in acute versus chronic virus–elicited memory CD8^+^ P14 T cells was interrogated from an existing ATAC-Seq dataset (21). Naive CD8^+^ P14 T cells were used as a control. Data were analyzed by Mann-Whitney *U* nonparametric test and are depicted as mean ± SEM. *P < 0.05, **P < 0.01, ****P < 0.0001.

Given the above results demonstrating that FcγRIIB is expressed and, therefore, could modulate the survival and/or function of CD8^+^ T cells during viral infection, we employed a mouse model of lymphocytic choriomeningitis virus (LCMV) infection to interrogate the effects of FcγRIIB signaling on CD8^+^ T cells during viral infection. Naive B6 mice were adoptively transferred with Thy1.1^+^ P14 TCR transgenic T cells (specific for the LCMV viral glycoprotein epitope gp33-41/D^b^) and were infected using either an acute (Armstrong [Arm]) or chronic (Clone 13 [cl-13]) model of LCMV (Figure 2, A and B). Results indicate that the frequency of Thy1.1^+^ P14 T cells that expressed FcγRIIB increased significantly among CD8^+^ T cells in the blood of both Arm- and cl-13–infected animals. However, we observed a significantly greater frequency of antigen-specific Thy1.1^+^ CD8^+^ P14 cells expressing FcγRIIB on days 7, 14, and 21 following Arm compared with cl-13 infection (Figure 2, C and D). To confirm that FcγRIIB expression on virus-specific CD8^+^ T cells was the result of gene expression and not trogocytosis, transcript levels of *Fcgr2b* RNA isolated from P14 cells from Arm-infected versus cl-13–infected mice on day 30 from an existing dataset (20) (GSE9650) were compared. Results show that the relative expression of *Fcgr2b* mRNA was elevated in both acute and chronic model–elicited memory CD8^+^ P14 cells as compared with naive P14 T cells; however, the chronic model–elicited CD8^+^ T cells exhibited significantly lower levels of *Fcgr2b* gene expression than the acute virus–elicited memory CD8^+^ T cells (Figure 2E). Furthermore, chromatin accessibility of the *Fcgr2b* locus in acute versus chronic model–elicited memory CD8^+^ P14 T cells was interrogated from an existing ATAC-Seq dataset (21). Interestingly, 7 regions of the *Fcgr2b* promotor were highly accessible in memory CD8^+^ T cells elicited via the acute model of LCMV but were relatively less accessible in CD8^+^ T cells elicited via the chronic model of LCMV (Figure 2F). These data show that virus-specific CD8^+^ T cells upregulate transcription and remodel the local chromatin of the *Fcg2rb* gene during an antiviral immune response; however, there is a range in the frequency of FcγRIIB-expressing cells within antigen-specific CD8^+^ T cell populations.

**Figure 3:**
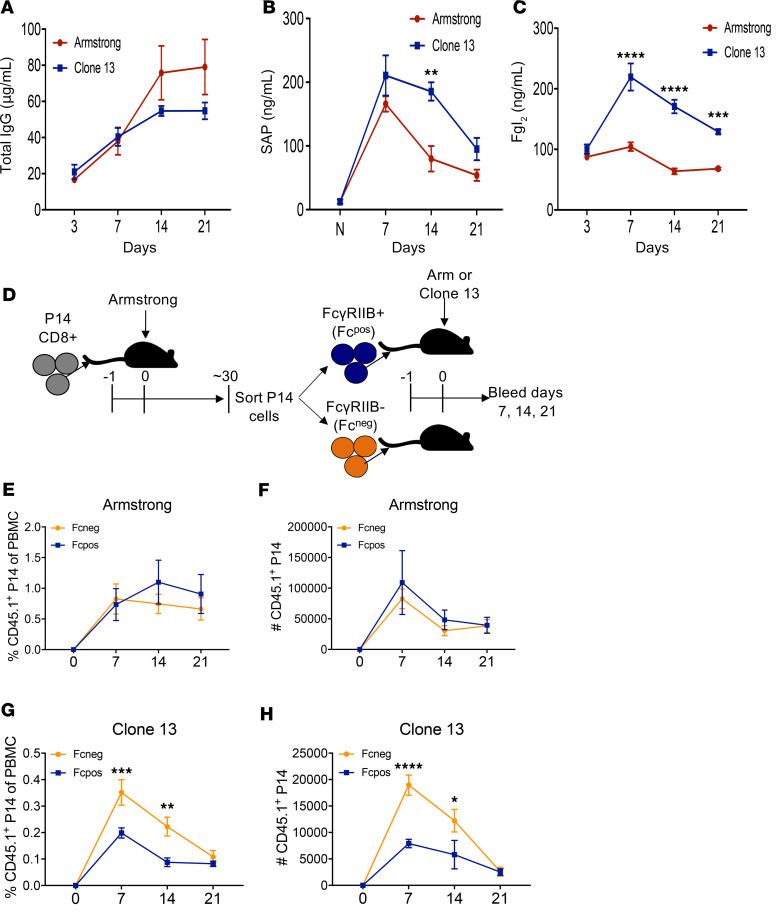
Transfer of FcγRIIB^+^ but not FcγRIIB^–^ CD8^+^ T cells into an Fgl2-rich environment results in loss of transferred T cells. (**A**–**C**) Naive B6 animals were infected either LCMV Arm or cl-13, and serum was analyzed on days 3, 7, 14, and 21 after infection for the concentration of total IgG (**A**), serum amyloid protein (SAP) (**B**), or Fgl2 (**C**). (**D**–**H**) Naive B6 animals received an adoptive transfer of 1 × 10^4^ congenically labeled CD45.1^+^ P14 T cells and were infected with LCMV Arm. On day 27, splenic CD45.1^+^CD8^+^ P14 T cells were sorted into FcγRIIB^+^ (Fc^pos^) and FcγRIIB^–^ (Fc^neg^) populations which were each transferred into naive hosts that were then infected with either Arm or cl-13 (**D**). (**E** and **F**) The frequency (**E**) and absolute number (**F**) of FcγRIIB^+^ (Fc^pos^) versus FcγRIIB^–^ (Fc^neg^) CD45.1^+^ CD8^+^ among PBMC in Arm-infected mice. (**G** and **H**) The frequency (**G**) and absolute number (**H**) of FcγRIIB^+^ (Fc^pos^) versus FcγRIIB^–^ (Fc^neg^) CD45.1^+^ CD8^+^ among PBMC in cl-13–infected mice. Data shown are *n* = 3–5 mice/group and are representative of 2 independent experiments. Data at each time point were compared by Mann-Whitney *U* nonparametric test and are depicted as mean ± SEM. *P < 0.05, **P < 0.01, ***P < 0.001, ****P < 0.0001.

One potential explanation for the observation of high frequencies of FcγRIIB^+^CD8^+^ T cells in the Arm infection model and low frequencies of FcγRIIB^+^ cells in the cl-13 infection model could be that the presence of the functional ligand for FcγRIIB in the cl-13–infected mice is driving apoptosis of FcγRIIB^+^ CD8^+^ T cells under these conditions, resulting in reduced frequencies of FcγRIIB^+^ CD8^+^ T cells. To address this possibility, plasma from Arm or cl-13–infected mice were analyzed for the concentration of 3 potential ligands of FcγRIIB: IgG (22), serum amyloid protein (SAP) (23), and Fgl2 (5, 12). Results indicate that plasma levels of IgG were significantly elevated on day 14 in mice infected with Arm (Figure 3A), and SAP was significantly elevated only on day 14 in mice infected with cl-13 (Figure 3B). In contrast, Fgl2 levels were consistently significantly elevated on days 7, 14, and 21 in mice infected with cl-13 as compared with those infected with Arm (Figure 3C). These data demonstrate an association of higher serum concentration of Fgl2 with lower frequencies of FcγRIIB^+^ CD8^+^ T cells during the course of viral infection.

### FcγRIIB^+^CD8^+^ T cells fail to expand in a Fgl2 replete environment.

To further distinguish between the possibility that the environment of cl-13 results in the preferential loss of FcγRIIB^+^CD8^+^ T cells versus the possibility that it simply does not induce the differentiation of FcγRIIB^+^CD8^+^ T cells, we performed an adoptive transfer experiment in which sorted FcγRIIB^+^ (Fc^pos^) and FcγRIIB^–^ (Fc^neg^) memory CD8^+^ P14 T cells obtained from d27 Arm-infected mice were transferred into naive hosts that were then infected with either Arm or cl-13 (Figure 3D). Results indicate that Fc^pos^ and Fc^neg^ CD8^+^ T cells exhibited similar kinetics and magnitude of expansion when adoptively transferred into Arm-infected animals (Figure 3, E and F), which have low serum concentrations of Fgl2 (Figure 3C). In contrast, Fc^pos^ and Fc^neg^ CD8^+^ T cells exhibited different fates within the Fgl2-rich cl-13 environment, in that the Fc^pos^ CD8^+^ T cells exhibited a competitive disadvantage in survival as indicated by a significantly lower frequency and cell number on days 7 and 14 following infection (Figure 3, G and H). These data provide evidence that FcγRIIB expression on CD8^+^ T cells limits their expansion and accumulation under conditions in which Fgl2 serum concentrations are high.

### Plasma concentration of Fgl2 is associated with CD8^+^ T cell lymphopenia in patients with COVID-19.

**Figure 4:**
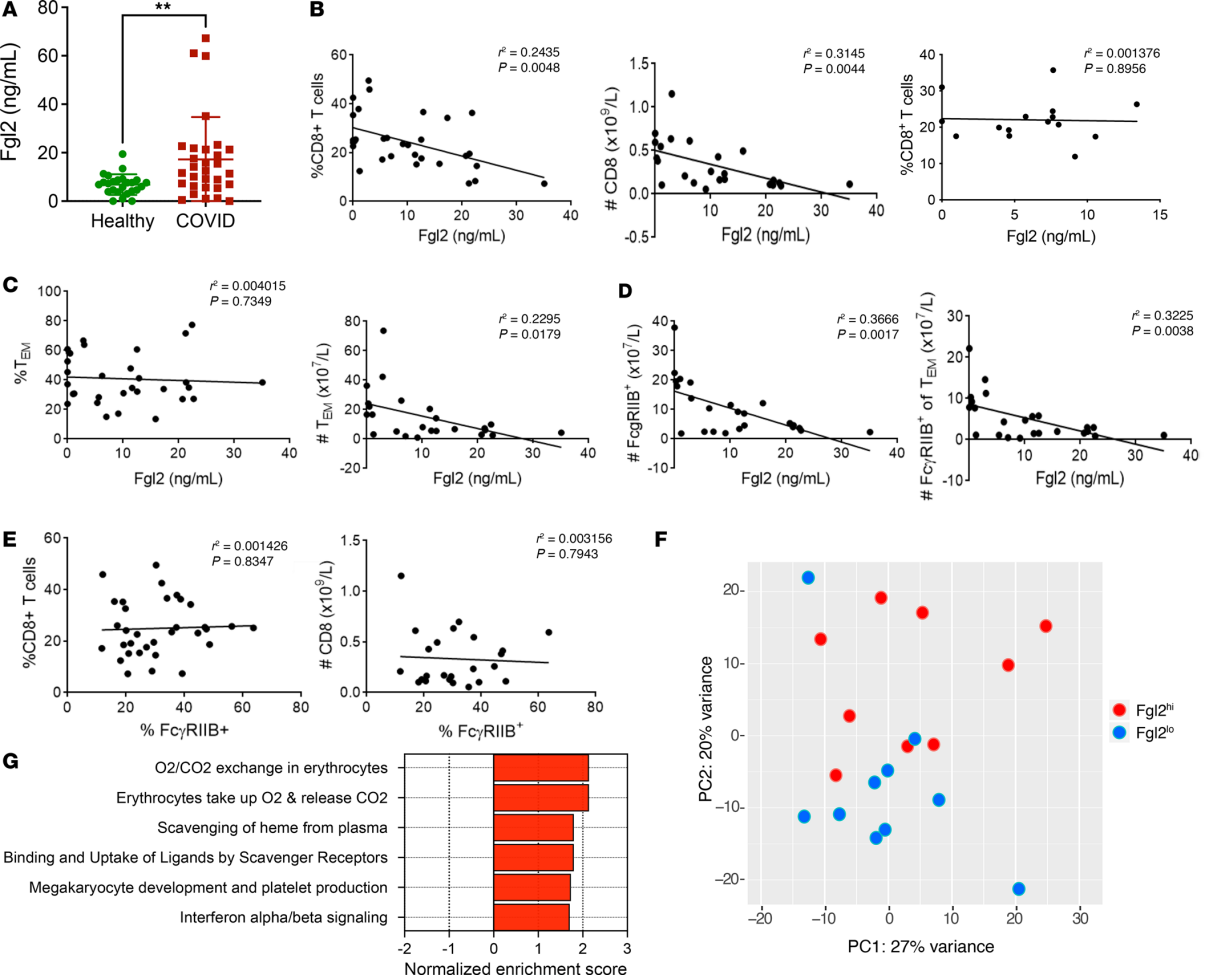
Fgl2 is increased in the serum of patients with COVID and is associated with decreased circulating CD8^+^ T cells. (**A**) Plasma obtained from *n* = 31 COVID^+^ patients (at 2–16, mean 8.9d following symptom onset) or *n* = 15 healthy patients was assayed for Fgl2 concentration (ng/mL) via ELISA. (**B**) PBMC was obtained from *n* = 31 patients testing positive for SARS CoV-2 admitted as inpatients at Emory University Hospital May-July 2020 (*n* = 31, Table 1) that were enrolled in an IRB-approved protocol and consented for blood draw. Blood samples were obtained 2-16 (mean 8.9) days after symptom onset. WBC were not available for *n* = 7 patients, thus all absolute count data represents *n* = 24 patients with COVID. Data were analyzed by Mann-Whitney *U* nonparametric test and are depicted as mean ± SEM. (**B**) Plasma concentration of Fgl2 (ng/mL) is plotted against the frequency of CD8^+^ cells among PBMC (left panel), the absolute number of CD8^+^ T cells within PBMC (right panel), or the frequency of CD8^+^ cells among PBMC in healthy controls (right panel). (**C**) Plasma concentration of Fgl2 (ng/mL) is plotted against the frequency of CD45RA^+^ CCR7^–^ Tem among CD8^+^ cells (left panel), and the absolute number of CD8^+^ CD45RA^+^ CCR7^–^ Tem within PBMC (right panel). (**D**) Plasma concentration of Fgl2 (ng/mL) is plotted against the absolute number of total FcγRIIB^+^CD8^+^ T cells within PBMC (left panel) and the number of FcγRIIB^+^CD8^+^CD45RA^+^CCR7^–^ Tem within PBMC (right panel). (**E**) Frequency of FcγRIIB^+^ among CD8^+^ T cells is plotted against the frequency of CD8^+^ cells among PBMC (left panel), and the absolute number of CD8^+^ T cells within PBMC (right panel). Data were compared by linear regression analysis. (**F** and **G**) PBMC obtained from *n* = 21 patients positive for SARS-CoV-2 was subjected to bulk RNA-Seq (*n* = 10 patients did not have material available for RNA-Seq). (**F**) PCA of differentially expressed genes (DEG) between patients with high plasma Fgl2 concentrations versus those with low plasma Fgl2 concentrations. (**G**) GSEA of DEGs (FDR < 0.1). The normalized enrichment scores of significant pathways (FDR < 0.1) in patients exhibiting high versus low plasma Fgl2 concentrations are plotted.

Given the above data demonstrating that FcγRIIB^+^CD8^+^ T cell numbers are diminished in an Fgl2-rich environment, we interrogated the expression of plasma Fgl2 in patients testing positive for SARS-CoV-2, and investigated the potential association of Fgl2 plasma levels with CD8^+^ T cell lymphopenia. Results demonstrate that plasma Fgl2 concentration was significantly upregulated in patients with COVID-19 as compared with healthy controls (*P* = 0.0045; Figure 4A). Fgl2 levels in individual patients were then plotted against frequencies and numbers of indicated immune cell subsets obtained via flow cytometry studies as described in Figure 1. Importantly, a highly significant, inverse relationship between the plasma concentration of Fgl2 and both the frequency (*P* = 0.0048) and absolute number (*P* = 0.0044) of total CD8^+^ T cells was observed in patients with COVID-19 (Figure 4B) that was not observed in healthy controls (*P* = 0.9; Figure 4B). A significant, inverse relationship was also observed between the plasma concentration of Fgl2 and the number of CD8^+^ CD45RA^+^ CCR7^–^ Tem (*P* = 0.0179; Figure 4C) Moreover, a significant, inverse relationship was observed between the plasma concentration of Fgl2 and the number of both total FcγRIIB^+^CD8^+^ T cells (*P* = 0.0017) and FcγRIIB^+^CD8^+^CD45RA^+^CCR7^–^ Tem (*P* = 0.0038; Figure 4D). In contrast, no correlation between the frequency of FcγRIIB^+^CD8^+^ T cells and either the frequency or number of total CD8^+^ T cells was observed (Figure 4E), demonstrating that FcγRIIB expression alone was not associated with CD8^+^ T cell lymphopenia.

Given the above association between increasing plasma Fgl2 concentration and CD8^+^ T cell lymphopenia, we next assessed transcriptomic profiles of PBMC isolated from patients who exhibited higher versus lower plasma concentrations of Fgl2. Because the median plasma Fgl2 concentration in SARS-CoV-2 patients was 9.153 ng/mL, we used this median value as the cutoff and designated patients with plasma concentrations of Fgl2 ≥ 9.153 ng/mL as Fgl2^hi^ and patients with plasma concentrations of Fgl2 < 9.153 ng/mL as Fgl2^lo^. Using this methodology, we identified 213 differentially expressed genes (DEG) between the Fgl2^hi^ versus Fgl2^lo^ groups at a FDR < 0.1. Principal component analysis revealed the variation in gene expression profiles between the Fgl2^hi^ versus Fgl2^lo^ patient cohorts (Figure 4F). Gene set enrichment analysis (GSEA) revealed significant enrichment (FDR < 0.1) in pathways associated with erythrocyte metabolism, megakaryocyte development and platelet production in patients exhibiting high levels of plasma Fgl2 (Figure 4G).

### Fgl2 derived from a CD8^+^ T cell source results in FcγRIIB-mediated regulation of CD8^+^ T cells.

**Figure 5:**
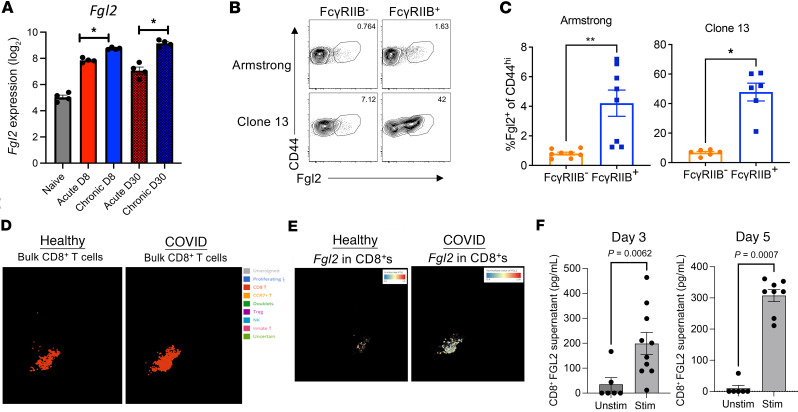
CD8^+^ T cells produce Fgl2 during viral infection in both mice and humans. (**A**) Fgl2 transcripts in CD45.1^+^CD8^+^ P14 T cells sorted by FACS that were isolated on either day 8 or day 30 from mice infected with either Arm (acute) or cl-13 (chronic) were assessed by bulk RNA-Sequencing and compared by 2-way ANOVA (24). (**B** and **C**) Fgl2 protein expression in CD45.1^+^CD8^+^ P14 T cells isolated from the spleens of either Arm-infected or cl-13–infected mice on day 27 after infection was assessed via intracellular cytokine staining following ex vivo restimulation with PMA/ionomycin. Representative flow cytometry plots are shown in **B**. Summary data from *n* = 3 mice/group are shown in **C**. Data depicted are mean ± SEM, using Mann Whitney *U* nonparametric test. Data are representative of 2 independent experiments. (**D** and **E**) Data mining experiment to identify expression of Fgl2 within CD8^+^ T cells from a single cell RNA-Seq data comparing healthy people and patients with COVID-19 (25). (**D**) The CD8^+^ T cell clusters in healthy versus patients with COVID are shown in red. (**E**) Expression of Fgl2 transcript within the CD8^+^ T cells in healthy versus patients with COVID is shown in white. (**F**) CD8^+^ T cells were FACS purified from healthy human PBMC and stimulated for 5 days with anti-CD3/anti-CD28. Supernatants were collected and Fgl2 concentration was assessed via ELISA. Data are depicted as mean ± SEM (*n* = 6–10), using Mann Whitney *U* test. *P < 0.05, **P < 0.01.

In light of the above results demonstrating that Fgl2 induces a loss of FcγRIIB^+^CD8^+^ T cells during viral infection, we next queried the cellular source of Fgl2. RNA-Seq data of sorted P14 CD8^+^ T cells isolated on either day 8 or day 30 from mice infected with either the acute (Arm) or chronic (cl-13) model of LCMV (24) demonstrate that Fgl2 could be produced by virus-specific CD8^+^ T cells themselves, with an increase in Fgl2 transcripts in CD8^+^ T cells elicited via the chronic versus acute viral infection model (Figure 5A). This was true for CD8^+^ T cells isolated on both day 8 and day 30 after infection (Figure 5A). To assess Fgl2 protein expression in the setting of both acute and chronic viral infection models, we stained splenocytes from day 27 after infection with anti-Fgl2 and anti-FcγRIIB. CD44^hi^FcγRIIB^+^CD8^+^ T cells elicited from both infections exhibited greater frequencies of Fgl2^+^ cells compared with CD44^hi^FcγRIIB^–^CD8^+^ T cells (Figure 5, B and C). Notably, 40% of CD44^hi^FcγRIIB^+^CD8^+^ T cells isolated from cl-13–infected mice expressed Fgl2, compared with only 1.5% of the CD44^hi^FcγRIIB^+^CD8^+^ T cells in Arm-infected mice (Figure 5, B and C). Taken together, these data suggest that Fgl2 cytokine can be produced by CD8^+^ T cells themselves. As an independent validation of this finding and to determine whether CD8^+^ T cells produce Fgl2 during COVID-19 in humans, we mined existing single-cell RNA-Seq data from Liao et al. comparing healthy people and patients with COVID-19 (25) and found that *Fgl2* transcript was upregulated in CD8^+^ T cells isolated from COVID-19 versus healthy controls (Figure 5, D and E). Finally, to verify that Fgl2 can be not just produced but also secreted by CD8^+^ T cells, we performed an in vitro assay in which CD8^+^ T cells were FACS purified (>90%) from healthy human PBMC and stimulated for 5 days with anti-CD3/ anti-CD28. Supernatants were collected, and Fgl2 concentration was assessed via ELISA. Results indicate that Fgl2 was detected in the supernatant of stimulated CD8^+^ T cells at both day 3 (Figure 5F) and day 5 (Figure 5F) after stimulation and was significantly upregulated compared with unstimulated CD8^+^ T cell cultures. In summary, these data demonstrate that CD8^+^ T cells can themselves both produce and secrete the immunosuppressive cytokine Fgl2 during viral infection in both mouse and humans.

## Discussion

The data above show that FcγRIIB can be upregulated in the course of viral infection, including SARS-CoV-2 in humans. We recently showed in models of transplantation and antitumor response that genetic deletion of FcγRIIB on CD8^+^ T cells resulted in reduced T cell apoptosis during the execution of the immune response (5–7). Our previous work also showed that, surprisingly, an antibody was not required for FcγRIIB-mediated induction of apoptosis on CD8^+^ T cells. Instead, we identified a role for the immunosuppressive cytokine Fgl2 in inducing FcγRIIB-mediated apoptosis in tumor-specific CD8^+^ T cells (5, 8). While a previous report implicated a role for IgG antibody in FcγRIIB-mediated control of CD8^+^ T cell immunity (9), the role of Fgl2 in FcγRIIB-mediated regulation of anti-viral CD8^+^ T cell immunity is not well elucidated. Prior work also showed that Fgl2 played a role in suppressing antiviral immunity in LCMV but concluded that it did so by binding to FcγRIIB expressed on antigen presenting cells (26). Our data extend these findings by showing that FcγRIIB can be expressed on CD8^+^ T cells in this model and that Fgl2 can function directly on the FcγRIIB^+^CD8^+^ T cell to inhibit accumulation. Moreover, the source of the ligand that results in FcγRIIB-mediated CD8^+^ T cell apoptosis was not clear. Here, we show that CD8^+^ T cells are a source of Fgl2 in both mouse and human samples.

The data presented here may be clinically relevant in that patients with severe COVID-19 have more profound CD8^+^ T cell loss as compared with patients with less severe COVID-19 (15, 16). Our data suggest that CD8^+^ T cell loss could be a major driver of loss of viral control, leading to persistent inflammation and tissue pathology. The data presented herein suggest that this CD8^+^ T cell loss could be mediated at least in part by the Fgl2 pathway, in that high levels of serum Fgl2 in the context of COVID-19 is associated with fewer FcγRIIB^+^CD8^+^ T cells. This hypothesis is supported by our data showing that Fgl2 level inversely correlates with both the frequency and number of peripheral CD8^+^ T cells in patients with COVID-19, as well as the number of both Tem and FcγRIIB^+^CD8^+^ T cells. However, it is certainly likely that additional factors also contribute to CD8^+^ T cell loss during COVID-19, as has been previously reported (27).

Previously published reports have demonstrated that inflammation in general can result in the production of Fgl2 by innate immune cells, as is observed in LCMV-infected animals and in patients with both cancer and inflammatory bowel disease (28–33). However, the conditions under which CD8^+^ T cells are driven to produce Fgl2 have not been well elucidated. Here, we show that FcγRIIB expression is associated with Fgl2 expression in an animal model of viral infection. However, the causal relationship between these 2 observations remains to be determined. In addition, we observed increased Fgl2 expression in CD8^+^ T cells in the setting of a chronic versus acute viral infection, suggesting that persistent exposure to antigen and/or inflammation may drive CD8^+^ T cell expression of Fgl2. Indeed, we recently showed that Fgl2 production is enriched in PD-1^+^ cells in a model of antitumor immunity, suggesting that T cell exhaustion programs may drive expression of Fgl2 (8). Because these exhausted CD8^+^ T cells can also express the Fgl2 receptor FcγRIIB, ligation of which induces CD8^+^ T cell apoptosis (5), production of Fgl2 provides a negative feedback loop by which an exhausted CD8^+^ T cell can induce its own death. We speculate that this pathway serves as a mechanism to prevent immune pathology during chronic viral infection and/or persistent T cell activation.

In addition to these generalized mechanisms of Fgl2 production, however, there may be a more specific way in which coronaviruses may elicit Fgl2 production, in that data from the early 2000s outbreak of SARS-CoV-1 show that Fgl2 is elevated in patients with SARS-CoV-1, and that the SARS-CoV-1 nucleocapsid (N) protein can directly induce transcription of the *hFgl2* gene via a C/EBPα binding partner (34). Moreover, GWAS studies of patients with SARS-CoV-1 showed a tight association between mutations in the human *Fgl2* gene and increased severity of disease (35). The only other candidate gene identified was CXCL10. Previous studies have also shown that mutations in the *CEBPA* gene resulted in reduced production of Fgl2 in human Tregs, macrophages, and monocytes in vitro, leading to the conclusion that Fgl2 production in immune cells is driven by the transcription factor C/EBPα (34).

Furthermore, it is now well established that patients with severe COVID-19 disease exhibit marked coagulopathy leading to fibrin/fibrinogen deposition in the lung and, in some cases, disseminated intravascular coagulopathy (36–38). Fgl2 in its membrane-bound form is a serine protease capable of directly cleaving prothrombin to thrombin, resulting in intravascular fibrin deposition (39). Thus, Fgl2 is an inflammatory mediator that can underly both coagulopathy and T cell lymphopenia, important features of severe COVID-19. However, it is important to note that the Fgl2/ FcγRIIB axis is not specific to SARS-CoV-2, as our work and the published studies of others have identified upregulation of both Fgl2 and FcγRIIB in other viral models (26, 40) as well as during transplantation and antitumor immunity (5–8, 19). Our RNA-Seq analysis comparing PBMC preparations isolated from patients with COVID-19 with high versus low plasma levels of Fgl2 identified an increase in genes associated with erythrocyte metabolism, megakaryocyte development, and platelet production in patients with high plasma Fgl2 levels. A gene signature associated with perturbations in erythrocyte membrane transport was also observed in an independent study of patients with COVID-19 (41) and is supported by the electron microscopy images showing polyhedrocytes in patients with COVID-19 (41). Taken together, these published clinical data together with our findings suggest that Fgl2 may mediate SARS-CoV-2 pathogenesis via 2 mechanisms: by increasing thrombosis and also by binding to FcγRIIB on T cells, resulting in T cell apoptosis and resultant lymphopenia. These data suggest that further investigation of the relationship between SARS-CoV-2 and Fgl2 is warranted.

## Methods

### Sex as a biological variable.

Our study examined male and female mice and humans, and similar findings are reported for both sexes.

### Human samples and collection.

Samples were derived from patients who tested positive for SARS-CoV-2 and were hospitalized at Emory University Hospital between May and July of 2020. Specific patient demographics are listed in Table 1. Blood samples were obtained 2–16 days (mean 8.9 days) after symptom onset via venipuncture and collected into CPT tubes (BD Biosciences). CPT tubes were spun for 30 minutes to separate RBC, WBC, and plasma. Plasma was harvested from the CPT first, avoiding the WBC layer, and placed in a conical tube for cryopreservation prior to analysis. PBMC were also cryopreserved and thawed later for batch analysis using flow cytometry. Healthy controls (*n* = 15) were 67% female, 60% Black/33% white/7% Asian, and ranged in age from 24 to 65, with a mean of 42 and median of 37. For analysis of Fgl2 in supernatant, PBMC from healthy donors were sorted by FACS on a BD FACSDiscover S8 Cell Sorter and stimulated for 5 days in vitro with Human T-Activator CD3/CD28 Dynabeads per manufacturer instructions.

### Mice and LCMV infections.

WT CD45.2^+^ C57BL/6J mice were obtained from The Jackson Laboratory aged 6–8 weeks old. Both female and male mice were used. All animals were housed in specific pathogen–free animal facilities at Emory University with infected mice housed in BSL2 animal facilities. CD45.1^+^ P14 mice and viral stocks of LCMV strains Arm and cl-13 were gifts from Rafi Ahmed (Emory University). For the LCMV Arm acute infection model, mice were injected with 2 × 10^5^ pfu i.p. For the LCMV cl-13 chronic infection model, mice were injected with 2 × 10^6^ pfu i.v.

### Adoptive transfers.

Spleens from CD45.1^+^ P14 transgenic mice were harvested and processed into single-cell suspensions. Splenocytes were counted using a Nexcelom Cellometer Auto T4 (Nexcelom Bioscience) and stained with CD8-BV786, CD4-PB, CD19-BV510, Vα2-FITC, and Vβ8-PE (BioLegend) to obtain the frequency of transgenic P14 Vα2^+^Vβ8^+^CD8^+^ T cells. Cells were resuspended in PBS, and 2 × 10^3^ P14 transgenic cells were adoptively transferred via i.v. into naive CD45.2^+^ C57BL/6J recipients 24 hours prior to infections with LCMV Arm or cl-13.

### Flow cytometry and sorting for adoptive transfers.

Peripheral blood was isolated via cheek puncture and lysed with High Yield Lysis Buffer (Invitrogen, HYL250). Spleens were harvested and processed into single-cell suspensions. Isolated cells were stained with CD8-BV786, CD4-PB, CD19-BV510, CD45.1-BV605, CD45.2-PE-Dazzle, and 2.4G2-biotin (BD Biosciences); streptavidin-APC, CD44-APC-Cy7, and PU.1-AF488 (BioLegend); anti-Fgl2 (Abnova, clone 6D9, conjugated to R-PE using Novus, 703-0010, according to manufacturer’s instructions), Vα2-FITC (BioLegend), and Vβ8-PE (BioLegend). For sorting, Arm-elicited P14 splenocytes were harvested on day 27 and extracellularly stained with CD8-BV786, CD4-PB, CD19-BV510, CD45.1-BV605, CD45.2-PE-Dazzle, 2.4G2-biotin, streptavidin-APC, and CD44-APC-Cy7 and sorted on CD45.1^+^CD8^+^ P14 cells that were either 2.4G2^+^ or 2.4G2^–^ to > 95% purity. In total, 10,000 cells were adoptively transferred into naive CD45.2^+^ C57BL/6J mice.

### SPADE analysis.

Traditional flow cytometry data were manually gated in FlowJo on CD3^+^CD8^+^ lymphocytes, and new FCS files were created containing only these gated events. The pregated data files were uploaded to Cytobank for automated analysis by spanning-tree progression analysis for density-normalized events (SPADE) (42). SPADE trees were generated using FcγRIIB, CD28, CD45RA, 2B4 (CD244), PD-1, DNAM, TIGIT, CCR7, and CD69 as the clustering channels. To simplify comparisons between previously healthy patients and patients with COVID-19, SPADE analysis utilized fold change groups with the data files from healthy patients set as the baseline samples. The resulting SPADE trees were colored by the parameter “percent total ratio log” or log_10_ (percent of total sample/average percent of total baseline) to more easily visualize differences between COVID-19 samples and the averaged healthy control samples. The percent of total CD8^+^ cells in each node was compared between healthy versus COVID-19 groups and phenotypically similar nodes demonstrating significant differences between healthy patients versus patients with COVID-19 were grouped into clusters.

### Intracellular cytokine staining.

Spleens were processed to cell suspensions, and blood underwent RBC lysis prior to staining. The samples were then stained for surface markers. Cells were permeabilized using a FoxP3/transcription factor kit (Invitrogen). For Fgl2 cytokine staining of P14 cells, splenocytes were ex vivo stimulated at 37°C for 4 hours with 0.4 μg/mL LCMV-gp_33-41_ (KAVYNFATM) peptide and 10 μg/mL GolgiPlug (BD Biosciences). After 4 hours, cells were processed and stained for phenotypic markers and Fgl2. Fgl2 antibody (Abnova) were conjugated to fluorophore with Lightning Link technology (or isotype) and validated using *Fgl2*^–/–^ splenocytes. All flow cytometry samples were acquired on a Fortessa or LSR II flow cytometer (BD Biosciences), and data were analyzed using FlowJo (Tree Star Inc.) and Prism (GraphPad Software). Absolute cell numbers were calculated using CountBright Beads (Invitrogen) according to the manufacturer’s instructions.

### Flow cytometry analysis for human samples.

Frozen PBMCs were thawed rapidly in a 37°C waterbath with agitation and immediately washed with complete RPMI media with 10% FCS and DNase. Cells were stained with the following fluorochromes: TIGIT-BUV395, CD8-BUV496, CD3-BUV737, CD4-BUV805, DNAM-BV421, CD14-BV510, CD19-BV510, PD-1-BD605, CD69-BV650, TIM-3-BV711, CCR7-BV786, LAG3-FITC, 41BB-PerCP-Cy5.5, FcγRIIB-PE (clone FLI8.26), CD40-PE-Dazzle/594, CD28-PE-Cy7, CD45RA–Alexa Fluor 700, and 2B4-APC-Cy7. For an FcγRIIB isotype control, a mouse, anti–human Ig2b, k-PE isotype was used. Cells were then stained with Live/dead aqua (Thermo Fisher Scientific), fixed (BD Cytofix/Cytoperm Fixation/Permeabilization Solution Kit), and run on the Fortessa Flow Cytometer.

### ELISAs.

To measure plasma and supernatant levels of Fgl2, the Fgl2 ELISA kit (BioLegend, 437807) was utilized according to the manufacturer’s instructions. To measure plasma levels of Total IgG, the Total IgG ELISA Kit (Thermo Fisher Scientific, 88-50400-22) was utilized according to the manufacturer’s instructions. To measure plasma levels of SAP, the SAP ELISA kit (Abcam, ab213888) was utilized according to the manufacturer’s instructions.

### RNA-Seq and data analysis.

RNA was extracted from thawed human participant PBMC using Qiagen RNeasy kits on an EZ1Advanced device according to manufacturer instructions. High-quality cDNA libraries were prepared from RNA extracts using Clontech SMARTseq v4 and sequenced on an Illumina NovaSeq6000 to a target depth of 18–20 million SR100 reads per sample by the Emory Non-Human Primate Genomics Core Facility. Gene expression was assessed by first generating gene counts using the fast RNA-Seq aligner STAR (Spliced Transcripts Alignment to a Reference) and human genome build (43).The R package DESeq2 (44) was used for normalization and differential expression analysis. Genes differentially expressed with a FDR < 0.1 were used as input for GSEA, which was performed using the Web-Based Gene Set Analysis Toolkit and the Reactome database (45). Pathways enriched at a FDR < 0.1 were plotted in Figure 4G.

### Reanalysis of publicly available datasets.

An independent single-cell RNA-Seq data of healthy patients and patients with COVID-19 were reanalyzed using BBrowser2 (BioTuring) (46). Liao et al. (25) performed single-cell RNA-Seq of 66,452 cells utilizing the 10x Chromium Single Cell Reagent Kit. After sequencing, the Cell Ranger Software Suite (v.3.1.0) was used for processing. Filtered gene code matrices were normalized, and UMAP was performed on top 2,000 variable genes for visualization in Seurat v.3. Statistical analysis was performed with GraphPad Prism 9. The dataset is available in the GEO database under accession no. GSE145926. Raw microarray data was downloaded from accession GSE9650 (20) and GSE41870 (24). Data processing and normalization was performed using the Affy R/Bioconductor package (47). ATAC-seq data (GSE83081) have been previously published (21) and were reanalyzed here. All other data display and analysis was performed using custom R/Bioconductor scripts.

### Statistics.

Statistical analyses were performed using GraphPad Prism software via Mann-Whitney *U* nonparametric test (for 2 groups) or 2-way ANOVA (for 3 or more groups). Association between T cell numbers and Fgl2 concentration was calculated using the Pearson correlation coefficient. Data are shown as mean ± SEM. A *P* value less than 0.05 was considered significant.

### Study approval.

The study design and conduct complied with the regulations on the use of human study participants and was conducted in accordance with the Declaration of Helsinki. Protocols were approved by Emory University’s IRB (IRB00022371 and STUDY00000510), and all donors gave written informed consent for the collection and use of the samples. Healthy controls were enrolled under an IRB-approved protocol (IRB00045821). This study was carried out in accordance with the recommendations in the *Guide for the Care and Use of Laboratory Animals* (National Academies Press, 2011). All animals were housed in specific pathogen–free animal facilities at Emory University. The protocol (PROTO201700558) was approved by the Emory University Institutional Animal Care and Use Committee (IACUC).

### Data availability.

All data contained within this manuscript are available in the Supporting Data Values. The RNA-Seq data presented in Figure 5 are publically available in the National Center for Biotechnology Information Genotype and Phenotype Database (NCBI dbGaP; accession no, phs003870.v1.p1).

## Author contributions

ABM designed, performed, and analyzed experiments and wrote the manuscript. MWA, KBB, CDM, KMM, MHW, CRL, and CDS designed, performed, and analyzed experiments and edited the manuscript. NR, CAM, and CSK designed experiments, assisted with the collection of human specimens, and edited the manuscript. MLF designed experiments, provided funding, analyzed data, and wrote the manuscript.

## Supplementary Material

Supplemental data

Supporting data values

## Figures and Tables

**Table 1:**
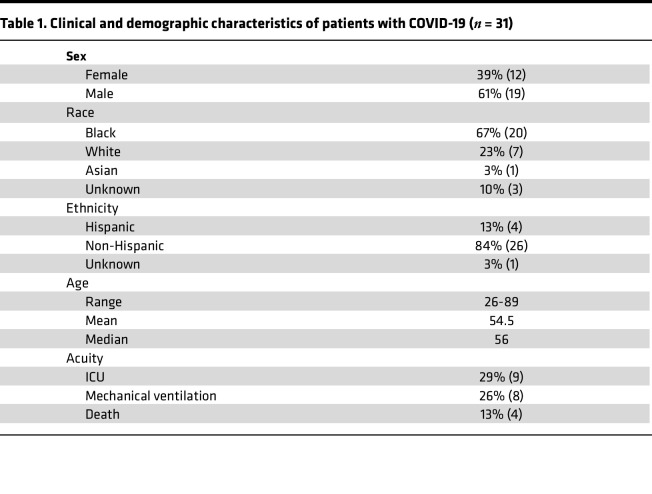
Clinical and demographic characteristics of patients with COVID-19 (*n* = 31)

## Version 1 04/08/2025

Electronic publication
