# Supplementary material for: Fgl2 regulates FcγRIIB+CD8+ T cell responses during infection
Source: JCI Insight. 2025 Apr 8;10(7):e186259. doi: 10.1172/jci.insight.186259 (PMC11981615; doi:10.1172/jci.insight.186259)
Supplement: Supplemental data [file jciinsight-10-186259-s138.pdf]

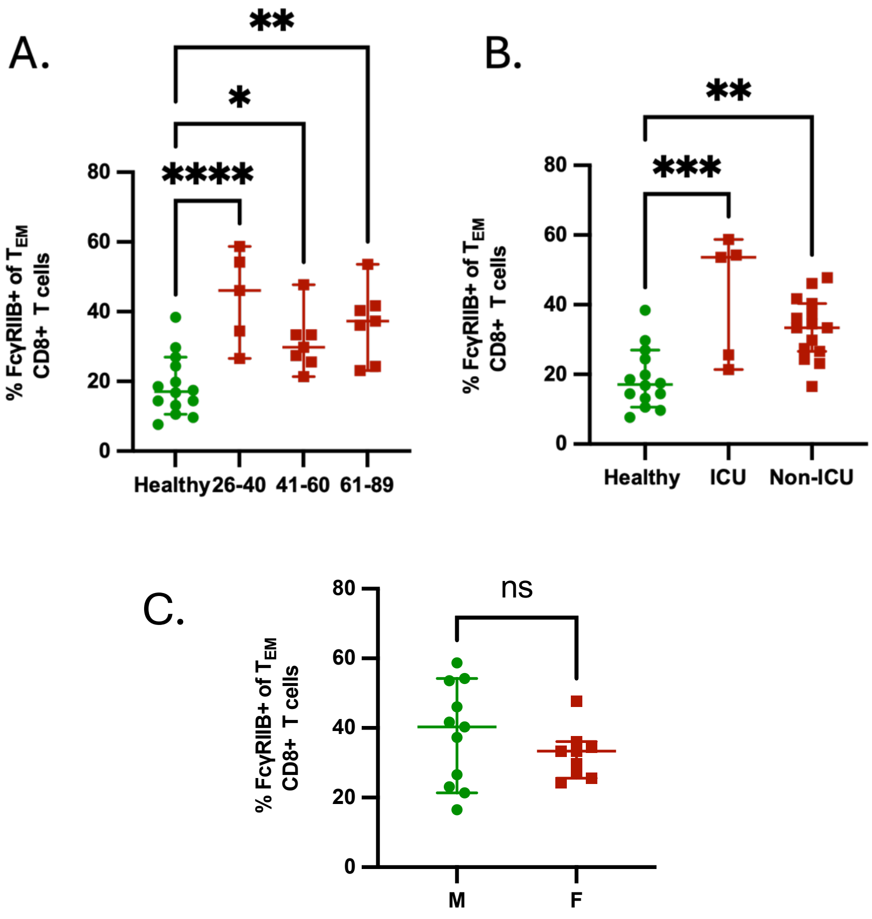

Supplemental Figure 1. Frequencies of FcγRIIB$^+$ among CD8$^+$ T cells are increased across age groups and disease severity. Patients testing positive for SARS CoV-2 admitted as inpatients at Emory University Hospital May-July 2020 and normal healthy controls were consented for blood draw and CD8$^+$ T cells were analyzed by flow cytometry. A, Summary data of the frequency of FcγRIIB$^+$ among CD8$^+$ T cells stratified by COVID patient age group. B, Summary data of the frequency of FcγRIIB$^+$ among CD8$^+$ T cells stratified by COVID patient disease severity (ICU or non-ICU). Data were analyzed by ANOVA with Dunnett's multiple comparisons post-test. C, Summary data of the frequency of FcγRIIB$^+$ among CD8$^+$ T cells stratified by sex. Data were analyzed by Mann-Whitney test. *$p<0.05$, **$p<0.01$, ***$p<0.001$, ****$p<0.0001$.
